# Influence of extracorporeal shockwaves on macrophage polarization in a 3D collagen matrix

**DOI:** 10.1016/j.reth.2025.06.003

**Published:** 2025-06-16

**Authors:** Diana Heimes, Moritz Große-Leege, Nadja Engel, Katharina Peters, Walburgis Brenner, Jürgen Brieger, Nadine Wiesmann-Imilowski, Peer W. Kämmerer

**Affiliations:** a Department of Oral and Maxillofacial Surgery, University Medical Center Mainz, Augustusplatz 2, 55131, Mainz, Germany; b Department of Oral and Maxillofacial Surgery, Facial Plastic Surgery, University Medical Center Rostock, Schillingallee 35, 18057, Rostock, Germany; c Department of Obstetrics and Gynecology, University Medical Center of the Johannes Gutenberg University Mainz, Langenbeckstraße 1, 55131, Mainz, Germany; d Department of Otorhinolaryngology, University Medical Center Mainz, Langenbeckstrasse 1, 55131, Mainz, Germany

**Keywords:** Macrophage polarization, ESWT, Extracorporeal shockwave therapy, 3D model, Collagen matrix

## Abstract

**Introduction:**

The host’s immune response determines the success of guided tissue regeneration. Extracorporeal shockwave therapy (ESWT) has been shown to reduce inflammation and improve wound healing. Nevertheless, its impact on macrophage polarization, as the first line of host defense, has not yet been elucidated. Therefore, this study aimed to investigate the effect of ESWT on macrophage polarization in a 3D collagen matrix model.

**Methods:**

Macrophages were isolated from human donor blood and differentiated into M1 macrophages. The cells were seeded into a collagen gel, and macrophages treated with ESWT (500 impulses, energy flux density 0.12 mJ/mm^2^) (+ESWT) were compared to non-treated cells (–ESWT). Furthermore, macrophages treated with 20 ng/μl IL-4 and 50 ng/μl M-CSF for M2-polarization in the gel (1.) or on a 6-well cell culture plate with Upcell™ surface (2.) as well as M1 macrophages cultured on a conventional cell culture dish (3.) served as reference cultures. Flow cytometry assessed polarization into M2 macrophages by measuring the expression of CD209, CD86, CD200R, and CD163.

**Results:**

Flow cytometry revealed no significant differences between the ESWT-treated (+ESWT) and untreated (–ESWT) macrophages for any of the markers. However, macrophages cultured in collagen gel (3) exhibited significantly higher CD200R expression rates than the other groups.

**Conclusions:**

The lack of significant differences in macrophage polarization following ESWT in this 3D model may be attributed to the absence of cell-cell interactions and other tissue structures that are present *in vivo*. Since the culturing environment significantly affected the expression of M2-polarization markers, with a significantly higher expression in collagen gel compared to cell culture plates, future studies should incorporate more complex co-culture systems or *in vivo* models to better simulate the clinical environment. Clinical evidence suggests that ESWT can effectively promote tissue healing and regeneration, indicating that the observed results may reflect limitations in the chosen model or cell type rather than the therapy itself, warranting further investigation.

## Highlights

• First study to assess ESWT effects on macrophage polarization in a 3D collagen matrix model.
• ESWT did not induce repolarization of M1 to M2 macrophages under the tested conditions.
• No significant changes in M2 markers (CD209, CD86, CD200R, CD163) after ESWT treatment.
• Results highlight the need for co-culture models to evaluate the cellular microenvironment.

## 1 Introduction

Tissue regeneration guided by biomaterials relies on these materials to activate and steer the body’s inherent healing mechanisms for tissue repair. A critical factor for success is the migration of cells into the biomaterials, supported by angiogenesis to sustain cell survival [1]. Of particular importance is the initial interaction between the biomaterials and the surrounding wound, which plays a pivotal role in determining the outcome of tissue regeneration [2,3]. The biological events that occur during this early phase, along with the subsequent processes of tissue formation, are critical for achieving favorable clinical outcomes, including successful integration and long-term stability [4,5]. Following augmentation, biomaterials are immediately exposed to serum proteins, initiating a cascade of interactions with host immune cells. These interactions, which are governed by the biomaterial’s surface properties and structural characteristics, result in the release of cytokines and growth factors, ultimately influencing the regenerative response [3,6].

The equilibrium between pro-inflammatory and anti-inflammatory activity ideally facilitates new tissue formation alongside biomaterial degradation. This immune response, particularly the role of various macrophage subtypes, has been shown to influence the foreign body reaction to biomaterials [7,8], with macrophages inducing angiogenesis and replacing neutrophils as key mediators of the inflammatory response during the early healing phase [9,10]. Following tissue injury, circulating monocytes are recruited from the bloodstream, where they differentiate into distinct phenotypes [11]. The literature primarily distinguishes between pro-inflammatory, antimicrobial M1 macrophages and anti-inflammatory, wound-healing M2 macrophages [10,12]. This simplified classification can be further subdivided based on function, with M2 macrophages categorized into subtypes M2a, M2b, M2c, and M2d [13,14]. However, the transitions between the described phenotypes are fluid. They should be viewed as a spectrum, as macrophages exhibit high plasticity and can shift between M1 and M2 profiles regardless of their initial polarization status [10,15]. In physiological wound healing, a polarization shift occurs: M1 macrophages dominate within the first three days post-injury, after which the wound environment transitions to a predominantly M2 macrophage population, peaking at seven days post-trauma [16,17].

In chronic wounds, persistent inflammation leads to excessive scarring, with macrophages pivotal in sustaining the chronic inflammatory response. Prolonged activity of M1 macrophages exacerbates inflammation, while enhanced M2 activity promotes tissue regeneration. However, excessive M2 polarization may result in fibrosis and excessive scar formation [18]. Thus, the balance between macrophage polarizations is crucial for optimal wound healing.

Targeted stimulation of macrophages, such as through extracorporeal shockwave therapy (ESWT), may enhance their tissue-regenerative properties, thereby improving healing outcomes. ESWT, which is an FDA-approved method for treating musculoskeletal disorders and various off-label applications [19], has historically been used to treat kidney stones [20]; today, radial and focused ESWT have also gained great scientific interest in the field of chronic wounds [21], nerve injury [22], erectile dysfunction [23], ischemic heart disease [24], and fracture non-union [25].

Radial extracorporeal shockwaves are typically generated by firing a projectile within a guiding tube that strikes a metal applicator on the patient’s skin. After reaching the tissue boundary, the waves spread non-invasively into the tissue [26].

In a recent review, Alshihri summarized translational applications of ESWT in dental medicine, reflecting the growing interest in ESWT use stemming from its non-invasiveness, low costs, and safety qualities, in addition to its proven regenerative, bio-stimulating aspects [27]. Strong evidence exists for improved wound healing in clinical settings, such as diabetic foot ulcers [28], keloids [29], burns [30], and even fractures non-unions [31]. This might be attributed to factors like increased tissue perfusion, immune modulation, and induction of cell proliferation [[32], [33], [34]]. Although extensive research has been conducted on the effects of ESWT on wound healing, the precise mechanisms driving its clinically observed anti-inflammatory effects remain unclear, warranting further investigation.

In one study, the inhibition of M1 macrophages and the increased activity of M2-associated genes have been demonstrated *in vitro* following ESWT [35]. This could at least partially explain the observed anti-inflammatory effect. However, a major limitation of previous *in vitro* studies lies in their experimental setup, which relied on transmitting shockwaves through an interface into a suspension. It can be anticipated that a significant portion of the energy is absorbed after passing the interface. Since evidence suggests that the cellular response to ESWT is largely determined by the complex interactions within a cellular network, involving impedance between different cell layers, intercellular connections, fluid dynamics, and chemotaxis, which are difficult to mimic *in vitro*, a key drawback of established *in vitro* models is the fluid character of cell suspensions.

Therefore, this study aimed to overcome these problems using a three-dimensional collagen matrix model including macrophages to analyze the effect of ESWT on macrophage polarization *in vitro*. The objective was to modify the initial pro-inflammatory M1 tissue response to a foreign material into an anti-inflammatory M2 response by applying extracorporeal shockwaves, to investigate whether persistent inflammatory reactions, such as those observed in foreign material rejection, can be prevented or treated by this approach.

## 2 Methods

### 2.1 Isolation of primary mononuclear cells (PBMC)

Monocytes for differentiation into macrophages were provided by the blood bank of the University Medical Center Mainz as buffy coats. 460 ml of human donor blood corresponded to 55–65 ml of buffy coat. The local ethics committee approved isolating primary blood mononuclear cells (PBMC) (State Medical Association Rhineland-Palatinate, No. 2021_16270). They were processed strictly anonymously without recording patient-related data and in accordance with the Declaration of Helsinki. Informed consent was obtained from each patient.

Isolation of PBMC was performed using density gradient centrifugation. For this, 15 ml of BioColl® (Bio&SELL GmbH, Feucht near Nürnberg, Germany) separation solution was initially added to each of the three 50 ml tubes. Subsequently, 20 ml of donor blood was slowly pipetted onto the separation solution, ensuring the two media did not mix. Centrifugation was performed for 30 min at 21 °C, 800×*g*, and with the break off.

Four layers formed in the tube through centrifugation: the top layer consisted of plasma, followed by a whitish layer of PBMCs. Below was the layer of the separation solution, and at the bottom were the erythrocytes. Using a 5 ml pipette, the PBMC layer was then collected and transferred into a pre-prepared, chilled washing solution for PBMCs (500 ml DPBS [Thermo Fisher Scientific Inc., Waltham, MA, USA] + 1 ml 0,5 M EDTA [Sigma-Aldrich, St. Louis, MO, USA]). The tubes were centrifuged for 6 min at 400×*g* and 6 °C. To retain the fragile cell pellet, 90 % of the supernatant was removed, and the cell pellet was resuspended in 50 ml of the washing solution. Following another round of centrifugation and supernatant removal, the pelleted cells were resuspended. This process was repeated until the cell pellet was firm and the supernatant was clear after centrifugation. The supernatant was removed, and the cells were resuspended in 40 ml of the washing solution.

Finally, the cell suspension was diluted at a 1:20 ratio with 0.4 % Trypan blue solution (Sigma-Aldrich, St. Louis, MO, USA). Counting of living cells was performed in a Neubauer counting chamber (Laboroptik Ltd., Lancing, UK) under the Leica DM2000 microscope (Leica Microsystems GmbH, Wetzlar, Germany) in at least two opposing squares. On average, 400,000,000 PBMCs were counted.

### 2.2 Plasma preparation

Five milliliters of human plasma from the buffy coat were transferred from each of the three 50 ml tubes into a 15 ml tube. This tube was incubated in a water bath at 56 °C for half an hour to heat-inactivate the contained proteins. Subsequently, the plasma was centrifuged again for 10 min at 1000×*g* and 6 °C; the supernatant was removed and transferred to a new 15 ml tube. The obtained plasma was used as an additive to the cell culture medium.

### 2.3 Isolation of monocytes

First, the adhesion solution was prepared. For this, RPMI 1640 medium (Thermo Fisher Scientific Inc., Waltham, MA, USA) was warmed in a water bath at 37 °C, and then 1 % GlutaMAX™ (Thermo Fisher Scientific Inc., Waltham, MA, USA) was added. The medium was incubated in a CO_2_ incubator at 37 °C, 5 % CO_2_, and 95 % humidity until use.

From the total cell count obtained, the required amount of cell suspension for further cultivation was calculated and then transferred to a new 50 ml tube. This was then centrifuged for 6 min at 400×*g* and 6 °C. The supernatant was discarded, and the cell pellet was resuspended in the appropriate adhesion solution. The cells were seeded into a 145 mm cell culture dish (Greiner Bio-One GmbH, Kremsmünster, Austria) for adhesion. At least 200,000,000 cells were resuspended in a 25 ml medium and transferred to the cell culture dish. The entire PBMC from a single donor was seeded onto a maximum of two cell culture dishes to ensure no cells were wasted. The dishes were then incubated for 40 min in a CO_2_ incubator at 37 °C, 5 % CO_2_, and 95 % humidity. During this time, primarily monocytes adhered to the cell culture dish.

The remaining PBMC, which did not adhere to the cell culture dish after incubation, was removed by gently rinsing five times in a circular motion with the adhesion solution. The solution was then discarded. Subsequently, 12.5 ml of pre-warmed (37 °C) RPMI 1640 medium was added to the cell culture dish and rinsed five times. This step was repeated until more adherent monocytes than PBMC were visibly observed under the Leica DMi1 microscope (Leica Microsystems GmbH, Wetzlar, Germany).

Subsequently, the RPMI 1640 medium was removed. Differentiation into M1-like macrophages was achieved by adding 50 ng/μl recombinant human GM-CSF (ImmunoTools GmbH, Friesoythe, Germany) to the differentiation medium for M1-like macrophages. 35 ml of this medium was added to each cell culture dish, followed by a CO_2_ incubator at 37 °C, 5 % CO_2_, and 95 % humidity for 5 days.

### 2.4 Differentiation of M1-like macrophages into M1 macrophages

After 5 days of incubation, the medium was removed, and the differentiation medium for M1 macrophages was prepared (RPMI 1640, GlutaMAX™ [1 %], human plasma [1 %], 50 ng/μl rh GM-CSF, 20 ng/μl rh INF-γ [ImmunoTools GmbH, Friesoythe, Germany]). Subsequently, 25 ml of this medium was pipetted into each cell culture dish, which was then transferred to a CO_2_ incubator and incubated at 37 °C, 5 % CO_2_, and 95 % humidity for 24 h.

### 2.5 Harvesting macrophages

After an incubation time of 6 days, the cell culture dishes were gently rinsed with a 5 ml pipette, and the medium was removed. Subsequently, 20 ml of Accutase® (Sigma-Aldrich, St. Louis, MO, USA) was added to each cell culture dish, followed by incubation for 1 h at 4 °C. After this period, 10 ml of RPMI 1640 was added, and the cells were gently detached from the bottom of the cell culture dishes using a spatula-shaped rubber scraper (Karl Hecht GmbH & Co. KG, Sondheim vor der Rhön, Germany). The resulting cell suspension was used to rinse the cell culture dish multiple times and transferred to a 50 ml tube.

An additional 12.5 ml of chilled (4 °C) RPMI 1640 was added to the cell culture dishes, and the dishes were checked for any remaining cells using the Leica DMi1 microscope. If cells remained adhered, the rinsing process was repeated. The tubes were then filled to 50 ml with RPMI 1640 and centrifuged for 6 min at 400×*g* and 6 °C. After removing the supernatant, the cell pellet was resuspended in 5 ml of RPMI 1640.

Living cells were counted using 10 μl of the cell suspension with 10 μl of Trypan blue solution in the Neubauer counting chamber.

### 2.6 Comparison groups with their respective cell culture conditions

• **“+ESWT”**: Cells cultured in collagen gel on a 12-well cell culture plate (Greiner Bio-One GmbH, Kremsmünster, Austria) and the addition of 50 ng/μl rh GM-CSF (ImmunoTools GmbH, Friesoythe, Germany), both to the gel and the medium, with a single application of shockwaves on day 7.
• **“– ESWT”**: Cells cultured in collagen gel on a 12-well cell culture plate without application of shockwaves and the addition of 50 ng/μl rh GM-CSF (ImmunoTools GmbH, Friesoythe, Germany), both to the gel and the medium.
• **“Positive Control Gel”**: Cells cultured in collagen gel on a 12-well cell culture plate with the addition of 20 ng/μl IL-4 (R&D Systems, Inc., Minneapolis, MN, USA) and 50 ng/μl rh M-CSF (ImmunoTools GmbH, Friesoythe, Germany), both to the gel and to the medium.
• **“Positive Control Cell Culture Plate”**: Cells cultured on a 6-well cell culture plate with Upcell™ surface (Thermo Fisher Scientific Inc., Waltham, MA, USA) from day 6 to day 8 with the addition of 20 ng/μl IL-4 and 50 ng/μl rh M-CSF (ImmunoTools GmbH, Friesoythe, Germany).
• **“Untreated”**: Differentiated M1 macrophages harvested from a 145 mm cell culture dish (Greiner Bio-One GmbH, Kremsmünster, Austria) on day 6, immediately before using these cells for the comparison groups listed above.

### 2.7 Preparation of collagen gel

The bovine collagen gel used (PureCol®, Advanced BioMatrix Inc., Carlsbad, CA, USA) was liquid at 4 °C. It started to cross-link as the temperature rose and a pH of 7.2–7.4 was reached, solidifying optimally at approximately 37 °C. Therefore, the gel was handled on ice until the desired solidification was achieved.

For the collagen gel preparation, 4 ml of collagen solution, 500 μl of tenfold concentrated RPMI 1640 (Sigma-Aldrich, St. Louis, MO, USA), and 50 ng/ml rh GM-CSF were mixed in a 15 ml tube using a 1 ml pipette. Since the gel cross-links ideally at a pH of 7.2–7.4, the pH was adjusted with NaOH and monitored with a pH meter during the initial preparation. The gel appeared pale yellow in acidic conditions and turned a deep pink under neutral pH. The amount of NaOH used (1280 μl) was recorded and used for further experiments, relying solely on the optical color control.

### 2.8 Preparation of collagen gel for **“Positive Control Gel”**

The collagen gel preparation for the positive control was conducted by adding 20 ng/μl rh IL-4 and 50 ng/μl rh M-CSF to the collagen gel instead of 50 ng/ml rh GM-CSF, which was prepared as described above. This control aimed to investigate the potential changes in the differentiation of M1 to M2a macrophages induced by IL-4 and M-CSF within the collagen gel and to compare these changes with the stimulus provided by shockwave treatment.

### 2.9 Transferring macrophages into the collagen gel

For the experimental setup, two 12-well cell culture plates were used; one for the group that received shockwave treatment and one for the other two gel collectives. For all three comparison groups, two wells were prepared. In each well, 1 ml of the collagen gel mixture containing one million M1 macrophages was distributed. The required amount of cell suspension from the harvested macrophages was centrifuged for 6 min at 400×*g* and 6 °C. The pelleted cells for the **“+ESWT”** and **“-ESWT”** collectives were then resuspended in 4 ml of the collagen gel mixture. The cell pellet for the **“Collagen Gel Positive Control”** was resuspended in 2 ml of the respective collagen gel mixture containing 50 ng/μl rh M-CSF, 20 ng/μl rh IL-4.

Subsequently, 1 ml of the cell-gel suspension was pipetted into each corresponding well. The remaining wells were each filled with 1 ml of DPBS.

**Fig. 1:**
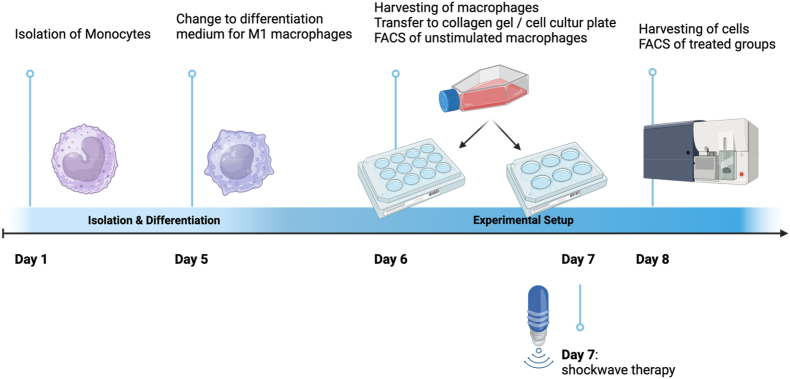
Timeline of the Experimental Setup. It was divided into two separate phases: I) the isolation and differentiation of the cells, and II) the execution of the experiment itself. At the beginning of phase 2, the macrophages were harvested from their cell culture plates and subsequently analyzed by FACS; this population represents the “untreated” group. The cells were then divided into four subgroups and prepared with the corresponding medium additives. The groups “+ ESWT” and “– ESWT” were cultured in gel on a 12-well cell culture plate with both, the culture medium and the gel, supplemented with 50 ng/μl rh GM-CSF. On day 8, a single application of ESWT was performed in the “+ ESWT” subgroup. For the “Positive Control Gel”, the cells were cultured in gel on a 12-well cell culture plate, with the addition of 50 ng/μl rh M-CSF and 20 ng/μl rh IL-4 to both the gel and the culture medium. The “Positive Control Cell Culture Plate” subgroup was cultured on a 6-well cell culture plate with the addition of 50 ng/μl rh M-CSF and 20 ng/μl rh IL-4 to the culture medium. Created with BioRender. com.

The gel was then solidified for 1 h at 37 °C, 5 % CO_2_, and 95 % humidity. Subsequently, 1 ml of M1-like-cell culture medium (RPMI 1640, GlutaMAX™ (1 %), human plasma (1 %), and 50 ng/μl rh GM-CSF was applied to the gel in the following groups: **“-ESWT”** and **“+ESWT”**. The **“Collagen Gel Positive Control”** received 1 ml of M2-cell culture medium (RPMI 1640, GlutaMAX™ (1 %), human plasma (1 %), 50 ng/μl rh M-CSF, 20 ng/μl rh IL-4). The experimental setup is presented in Fig. 1.

### 2.10 Cultivation of the macrophages on a cell culture plate

For the **“Positive control cell culture plate,”** 3,000,000 of the cells previously differentiated into M1 macrophages were subsequently cultured on a Nunc™ 6-well cell culture plate with Upcell™ surface for two days and differentiated into M2 macrophages with the addition of 50 ng/μl rh M-CSF and 20 ng/μl rh IL-4. This should provide the opportunity to compare the potential of the macrophage responses to the different stimuli in the collagen gel with those of conventional cultivation on cell culture plates, without the influence of forced detachment, for example, by Accutase or a cell scraper.

The cells were resuspended in an M2-cell culture medium previously heated to 37 °C immediately after removal from the cell culture plate. Each well received a total of 500,000 cells in a 2 ml medium. The cells were then incubated for two days in a CO_2_ incubator at 37 °C, 5 % CO_2,_ and 95 % humidity.

### 2.11 Extracorporeal shockwave therapy (ESWT)

**Fig. 2:**
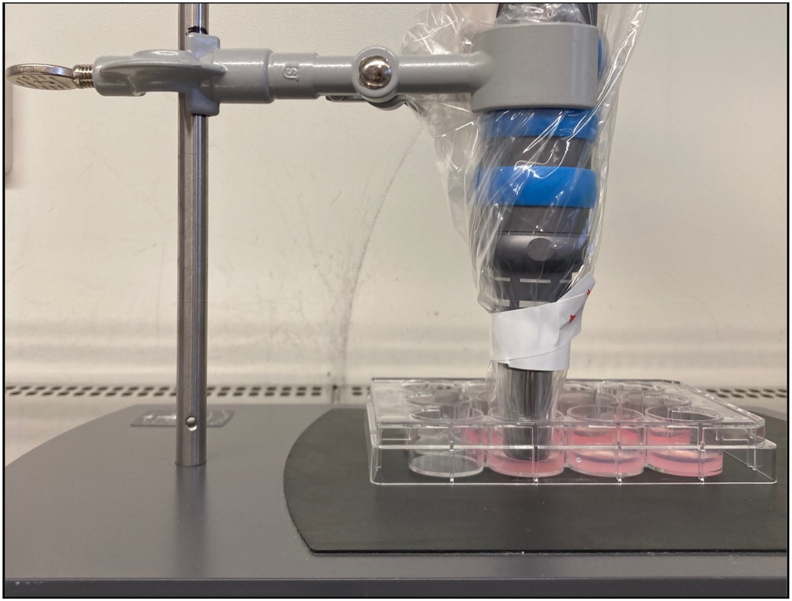
Extracorporeal shockwave application. For this purpose, the applicator head and the handpiece were covered with a sterile disposable cover. The handpiece was clamped into a stand so that the applicator head comes into direct contact with the gel. After the removal of the medium, shockwaves were applied by placing the metal applicator of the handpiece on the collagen gel. The **“+ESWT”** group was treated with 500 impulses of an energy flux density of 0.12 mJ/mm^2^ (3 bar air pressure, 5 Hz frequency).

Shockwaves were applied to the collagen gel using the Swiss DolorClast® Classic (EMS Electro Medical Systems SA, Switzerland). First, the tip of the applicator head was coated with ultrasound gel. Subsequently, the handpiece and applicator head were covered with a sterile single-use ultrasound cover. The handpiece was then secured in a stand so that the applicator head made direct contact with the collagen gel (Fig. 2). This setup prevented unwanted movements during the experiment. Additionally, the cell culture plate was placed on a rubberized surface to prevent the reflection of the shockwaves. After removing the medium, shockwaves were applied by placing the metal applicator of the handpiece on the collagen gel. The “**+ESWT”** group was treated with 500 impulses of an energy flux density of 0.12 mJ/mm^2^ (3 bar air pressure, 5 Hz frequency) as previously described [32]. Afterwards, 1 ml of M1-like-cell culture medium was added to the gel, and the wells were then transferred to a CO_2_ incubator and incubated at 37 °C, 5 % CO_2_, and 95 % humidity for 24 h.

### 2.12 Harvesting of the cells

For removal of the macrophages from the collagen gel, 1 ml collagenase I solution (100 mg collagenase type I 125 U/mg [Worthington Biochemical Corporation, Lakewood, NJ, USA], 10 ml HBSS [Thermo Fisher Scientific Inc., Waltham, MA, USA]) and 1 ml collagenase II solution (100 mg collagenase type II, 125 U/mg [Worthington Biochemical Corporation, Lakewood, NJ, USA], 10 ml HBSS) were mixed with 8 ml DPBS. To reduce cell activity, the harvesting procedure was performed at 4 °C.

The gel from one well was carefully transferred to a 15 ml tube using a pipette and mixed with 5 ml DPBS. Centrifugation was then carried out at 4 °C and 300×*g* for 5 min. The supernatant was carefully discarded using a pipette, and 5 ml DPBS was added again. Centrifugation was carried out under the same parameters. Then, 1 ml of the digestion solution was pipetted into the gel and carefully mixed. The suspension was transferred to sterile 2 ml Eppendorf tubes, which were then incubated in a thermomixer at 37 °C and 400 rpm for 40 min. After 10, 20, and 30 min, the tubes were carefully swirled by hand. At the end of the incubation period, the solution was transferred to a 50 ml tube, which was centrifuged at 4 °C and 400×*g* for 5 min. After removal of the supernatant, the pelleted cells were resuspended with 1 ml DPBS and centrifuged again at 4 °C and 400×*g* for 5 min. The supernatant was then removed again, and the cell pellet was resuspended in 500 μl Fluorescence Activated Cell Sorting (FACS) buffer (DPBS, 1 mM EDTA, human albumin (0.5 %), 10 μg/ml Privigen® [CSL Behring GmbH, Marburg, Germany]) and subsequently counted.

After two days of incubation, the 6-well cell culture plate was placed under a safety bench at room temperature for 30 min. The wells were then carefully rinsed with a 1 ml pipette, and the cell suspension was transferred to a 50 ml tube. Then, 1 ml RPMI 1640 was added to each well, and the cell culture plate was checked for cell residues under the Leica DMi1 microscope. If any remaining cells were found, the plate was rinsed 5 to 10 times with the existing medium. The cell suspension was then also transferred to the tube. The cell culture plate was checked again for cell residues and, if necessary, the rinsing process was repeated. The tube was centrifuged at 400×*g* and 6 °C for 6 min. Finally, the supernatant was removed; the cell pellet was resuspended in a 500 μl FACS buffer, and the cells were counted.

### 2.13 Flow cytometry

The differentiation stage of the macrophages was determined using flow cytometry. For this purpose, the cells were stained with fluorescence-labeled antibodies and then measured with the FACS CantoTM II cytometer (Becton, Dickinson and Company, Franklin Lakes, NJ, USA).

Three measurements were performed with each sample: a negative control (50 μl FACS-buffer), a mix of all the antibodies used (37.5 μl FACS-buffer, 2.5 μl of each antibody CD45, CD86, CD163, CD200-R, CD209, 1 μl SYTOX® Blue), and additional individual staining of each antibody used (37.5 μl FACS-buffer, 2.5 μl of antibody, Table 1).

**Table 1:** Material used for FACS analysis.

Antibody	Fluorescent Labeling	Manufacturer	Staining Goal
Anti-human CD45	PerCP	BioLegend®, San Diego, CA, USA	Leucocytes
Monoclonal CD86 (B7-2)	PE	Thermo Fisher Scientific Inc., Waltham, MA, USA	M1-phenotype
Anti-human CD163	Brilliant Violet 605	BioLegend®, San Diego, CA, USA	M2-phenotype
Anti-human CD200R	PE/Cyanine7	BioLegend®, San Diego, CA, USA	M2-phenotype
Anti-human CD209	FITC	BioLegend®, San Diego, CA, USA	M2-phenotype
SYTOX® blue dead cell stain	450/50-A	Thermo Fisher Scientific Inc., Waltham, MA, USA	Live-dead Discrimination

The groups **“Positive Control Cell Culture Plate,” “+ESWT,” “–ESWT,”** and **“Positive Control Gel”** were first stored on ice and then centrifuged at 400×*g* and 6 °C for 6 min. After removal of the supernatant, the cell pellet was resuspended in FACS buffer so that the cells were present in a concentration of 1 million cells in 1 ml of FACS buffer. 200,000 cells were required for each FACS measurement. Thus, 200 μl of the cell suspension was transferred to one well of a 96-well culture plate (U-shape) for each measurement and then centrifuged at 400×*g* and 6 °C for 3 min.

After removing the supernatant, the cells were resuspended in the different staining solutions (2.5 μl antibody in 47.5 μl FACS buffer for individual staining of antibodies and 2.5 μl of each antibody in 37.5 μl FACS buffer for the antibody mix) and incubated for 20 min at 4 °C. Subsequently, 150 μl FACS buffer was added to each well and centrifuged at 400×*g* and 6 °C for 3 min. The supernatant was removed again, and the pelleted cells were resuspended in 200 μl FACS buffer. The suspensions were then transferred to a round-bottom tube and stored on ice until measurement. Before the measurement, 1 μl SYTOX® Blue was added to the antibody mix.

Each sample was homogenized in a vortex before measurement. Then, 10,000 cells per sample were measured.

Using forward scatter (FSC) and side scatter (SSC), the cells were classified according to their size and granularity, and only CD45-positive macrophages were included in the further analysis. This procedure was performed for both the **“Untreated”** group and the other groups **(“+ESWT”, “–ESWT”, “Positive Control Gel”, “Positive Control Cell Culture Plate”**). Only those cells that met this requirement were analyzed further. With the help of SYTOX® Blue, the cells could be divided into live and dead. Only CD45-positive and SYTOX® Blue-negative cells of the live populations were considered for the analysis. The medians of the fluorescence signal of the living populations of the individual antibodies (CD86, CD163, CD200-R, CD209) of the FACS data were analyzed.

### 2.14 Statistics

The primary endpoint of this study was to determine whether ESWT could induce a shift in macrophage polarization from the M1 phenotype to M2 in a collagen gel model. To test the ability of M1 macrophages to change the polarization into M2 macrophages, cells seeded in collagen gel were further treated with IL-4 and M-CSF. The comparison between ESWT-treated cells and those being polarized using IL-4 and M-CSF was a secondary outcome of this study. To quantify the effect of the culture environment, M1 macrophages were either cultured in collagen gel or on a cell culture plate and polarized using IL-4 and M-CSF. The effect of the cultural environment served as a secondary outcome as well. Finally, all data were compared to the untreated cells, which were set as a negative control.

The statistical analyses were performed using SPSS version 24 for Windows (IBM, Armonk, NY, USA); graphics were generated using Microsoft® Excel Version 16.40 for Macintosh (Microsoft Corporation, Washington, DC, USA). To analyze the differences between the measured values, normality (Shapiro-Wilk) and homogeneity of variance tests (Levene Statistic) were performed first to check the conditions for the subsequent analysis. The data were analyzed using a one-way analysis of variance (ANOVA) to assess differences between groups. When significant differences were detected, Tukey’s post hoc test was applied to perform pairwise comparisons. *P*-values <0.05 were considered statistically significant. Results are presented as mean ± standard deviation (SD) unless otherwise stated.

## 3 Results

### 3.1 Analysis of flow cytometry

Flow cytometry was used to measure the median fluorescence intensity (MFI) values for the antibodies (CD209, CD86, CD200R, CD163).

### 3.2 **+ESWT** versus **–ESWT**

The comparison of cells cultured in collagen gel with and without ESWT should provide insight into the impact of ESWT on M1 macrophages.

The analysis showed that both groups had similar surface marker expressions for the four investigated markers (Table 2, Table 3, Table 4, Table 5). Using unpaired t-tests, no statistically significant differences were found between the treatment with or without shockwave therapy for any of the investigated surface markers (CD209: *p* = 0.703; CD86: *p* = 0.912; CD200R: *p* = 0.880; CD163: *p* = 0.919).

**Table 2:** MFI values of CD209 expression measured by flow cytometry.

Group	*n*	MFI	SD
+ESWT	10	306.00	117.33
–ESWT	10	289.40	67.65
Untreated	9	324.00	88.29
Positive control gel	6	249.67	37.27
Positive control cell culture plate	3	412.00	251.72

**Table 3:** MFI values of CD86 expression in flow cytometry.

Group	*n*	MFI	SD
+ESWT	10	9537.50	5548.96
–ESWT	10	9810.70	5330.32
Untreated	9	12660.89	3089.97
Positive control gel	6	8785.83	4224.21
Positive control cell culture plate	3	63451.33	22403.55

**Table 4:** MFI values of CD200R expression measured by flow cytometry.

Group	*n*	MFI	SD
+ESWT	10	3278.60	717.33
–ESWT	10	3232.00	575.39
Untreated	9	2347.67	944.23
Positive control gel	8	6595.67	1785.95
Positive control cell culture plate	3	2385.33	281.43

**Table 5:** MFI values of CD163 expression measured by flow cytometry.

Group	*n*	MFI	SD
+ESWT	9	364.11	111.13
–ESWT	9	369.56	111.43
Untreated	9	359.00	139.89
Positive control gel	7	333.83	119.62
Positive control cell culture plate	3	311.00	21.66

### 3.3 Positive control gel versus positive control cell culture plate

To evaluate the influence of the culturing method on the macrophages, cells cultured in collagen gel were compared to the conventional culturing on a cell culture plate with an Upcell™ surface. In both groups, the cells were stimulated with IL-4 and M-CSF to promote the differentiation from M1 to M2a macrophages, as shown in previous studies [36,37].

The MFI values for CD209 (*p* = 0.141) and CD163 (*p* = 0.760) showed no significant difference between the groups. However, the CD86 expression was found to be statistically significantly higher in the **“Positive control cell culture plate”** compared to the **“Positive control gel”** (*p* < 0.001). Contrarily, the expression of CD200R was significantly higher in macrophages cultured in collagen gel than those cultured on a cell culture plate (*p* = 0.006). However, when evaluating these results, the small sample size in the **“Positive control cell culture plate”** and the large standard deviations must be taken into account (Table 2, Table 3, Table 4, Table 5).

### 3.4 Expression of CD209

**Fig. 3:**
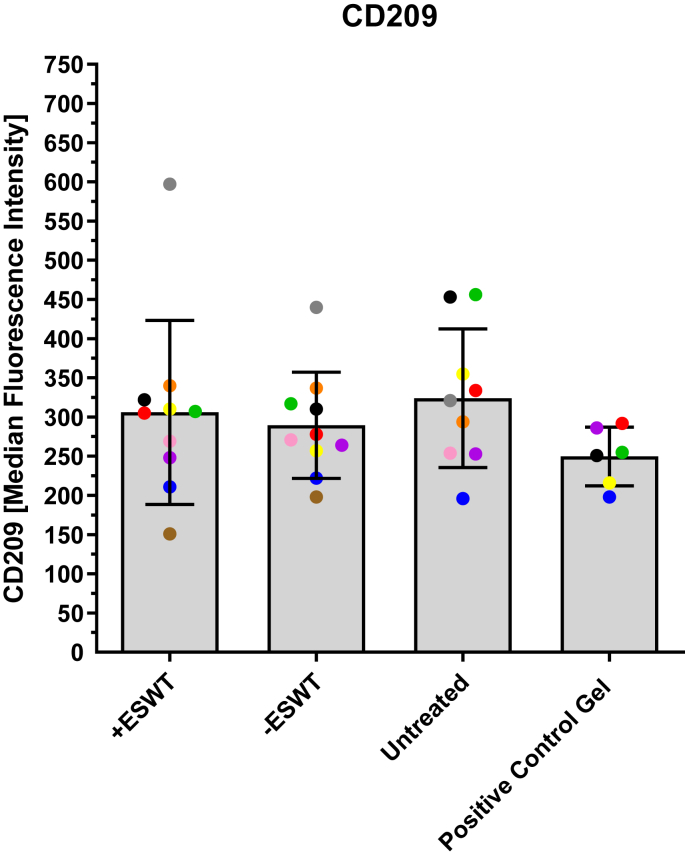
Overview of MFI values of the CD209 expression measured by flow cytometry. The MFI values of the CD209 expression are presented as a boxplot (median and interquartile range) and divided into four subgroups based on the type of treatment: **“+ESWT”** (*n* = 10), **“–ESWT”**(*n* = 10), **“Untreated”** (*n* = 9), and **“Positive Control Gel”** (*n* = 6). Individual data points are represented as dots in the bar chart. Macrophages from the same patient are consistently color-coded across all images. No statistically significant differences could be observed between the groups (**“–ESWT”** vs. **“+ESWT”**: *p* = 0.703; **“+ESWT”** vs. **“Untreated”**: *p* = 0.713; **“+ESWT”** vs. **“Positive Control Gel”**: *p* = 0.278; **“–ESWT”** vs. “**Untreated”**: *p* = 0.348; **“–ESWT”** vs. **“Positive Control Gel”**: *p* = 0.211; **“Untreated”** vs. **“Positive Control Gel”**: *p* = 0.075).

Fig. 3 and Table 2 show the MFI values of the CD209 expression measured by flow cytometry. The data of the individual groups differ only slightly from each other. The **“Untreated”** population had the highest values (324.00 ± 88.29), followed by the **“+ESWT”** group (306.00 ± 117.33) and the **“–ESWT”** group (289.40 ± 67.65). The **“Positive Control Gel”** showed the lowest values (249.67 ± 37.27). However, there were no statistically significant differences between the groups (*p* > 0.05).

### 3.5 Expression of CD86

**Fig. 4:**
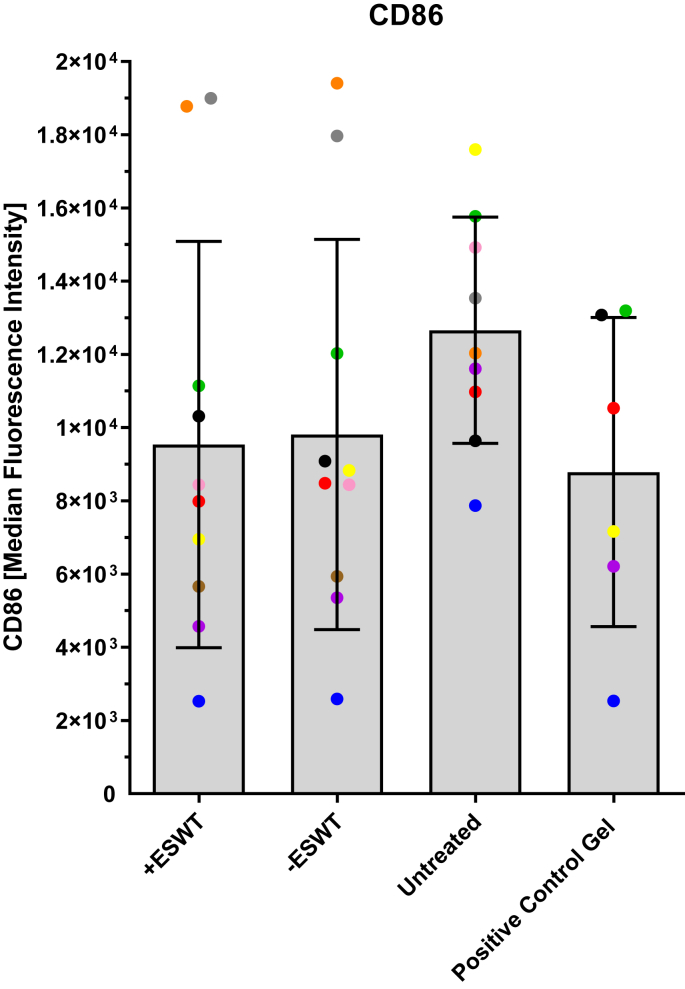
Overview of MFI values of CD86 expression measured by flow cytometry. The MFI values of the CD86 antibody in flow cytometry are presented as a boxplot (median and interquartile range) and divided into four subgroups based on the type of treatment: **“+ESWT”** (*n* = 10), **“–ESWT“** (*n* = 10), **“Untreated”** (*n* = 9), and **“Positive Control Gel”** (*n* = 6). Individual data points are represented as dots in the bar chart. Macrophages from the same patient are consistently color-coded across all images. No statistically significant differences could be observed between the groups **(“–ESWT”** vs. **“+ESWT”**: *p* = 0.912; **“+ESWT”** vs. **“Untreated”**: *p* = 0.154; **“+ESWT”** vs. **“Positive Control Gel”**: *p* = 0.780; **“–ESWT”** vs. **“Untreated”**: *p* = 0.178; **“–ESWT”** vs. **“Positive Control Gel”**: *p* = 0.695; **“Untreated”** vs. **“Positive Control Gel”**: *p* = 0.060).

Fig. 4 and Table 3 show the MFI values of the CD86 expression measured flow cytometry. Higher MFI values were measured for the **“Untreated”** group (12660.89 ± 3089.97) compared to the **“–ESWT”** group (9810.70 ± 5330.32), **“+ESWT”** group (9537.50 ± 5548.96), and **“Positive Control Gel”** group (8785.83 ± 4224.21). There were no statistically significant differences between the groups (*p* > 0.05).

### 3.6 Expression of CD200R

**Fig. 5:**
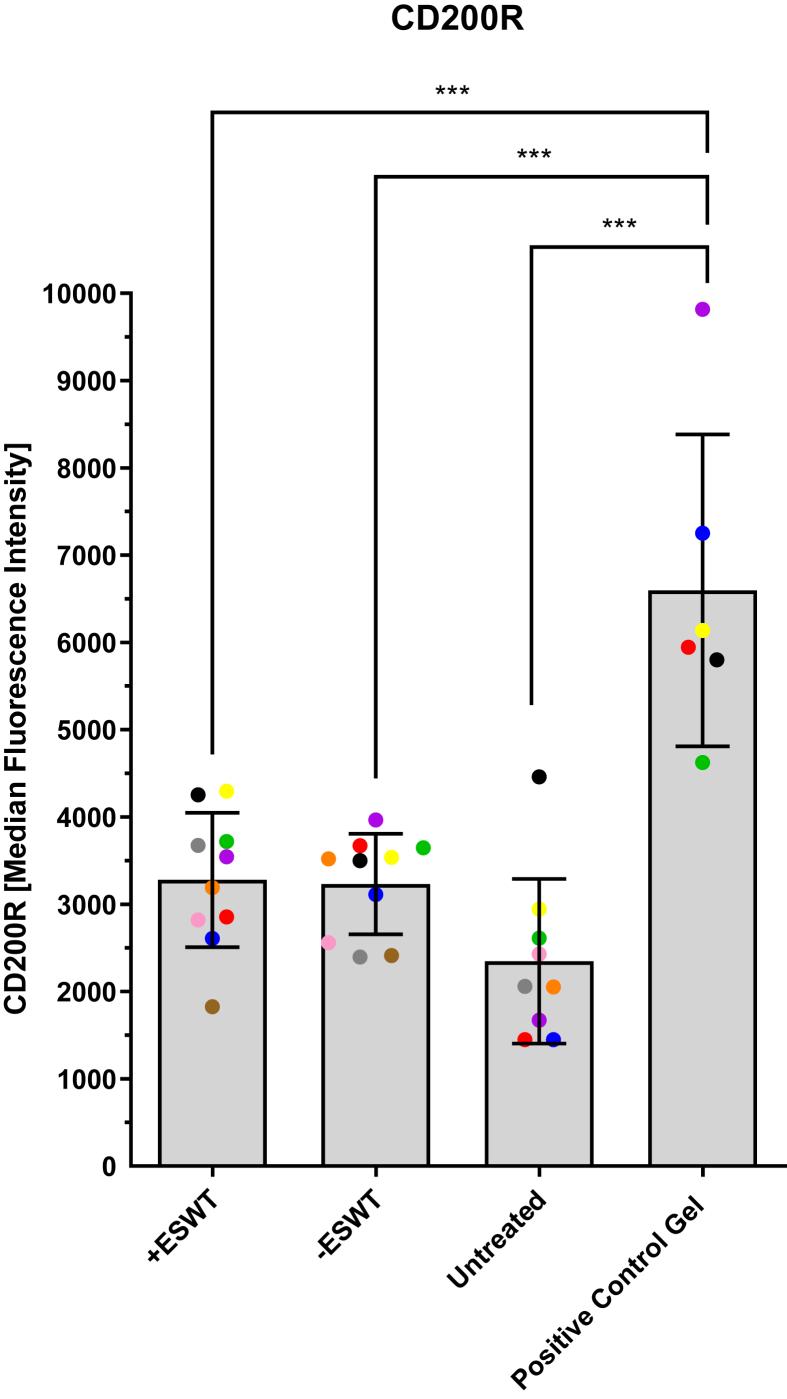
Overview of MFI values of CD200R expression measured by flow cytometry. The MFI values of the CD200R antibody in flow cytometry are presented as a boxplot (median and interquartile range) and divided into four subgroups based on the type of treatment: **“+ESWT”** (*n* = 10), **“–ESWT “**(*n* = 10), **“Untreated”** (*n* = 9), and **“Positive Control Gel”** (*n* = 6). Individual data points are represented as dots in the bar chart. Macrophages from the same patient are consistently color-coded across all images. Statistically significant differences could be observed for the following groups: **“+ESWT”** vs. **“Untreated”**: *p* = 0.030; **“+ESWT”** vs. **“Positive Control Gel”**: *p* < 0.001; **“–ESWT”** vs. **“Untreated”**: *p* = 0.023; **“–ESWT”** vs. **“Positive Control Gel”**: *p* < 0.001; **“Untreated”** vs. **“Positive Control Gel”**: *p* < 0.001). No significant differences could be observed between the **“–ESWT”** and the **“+ESWT”** groups (*p* = 0.880). ∗∗∗*p* < 0.001, ∗*p* < 0.05.

Fig. 5 and Table 4 show the MFI values of the CD200R expression measured flow cytometry. The highest MFI values were recorded in the **“Positive Control Gel”** group (6595.67 ± 1785.95) , followed by the **“+ESWT”** group (3278.60 ± 717.33) and the **“–ESWT”** group (3232.00 ± 575.39), which showed similar results. The measured values for the **“Untreated”** cells (2347.67 ± 944.23) were lower than those of the comparison groups. Statistically significant higher values could be observed in the **“Positive Control Gel”** group compared to the **“Untreated”** cells, the **“–ESWT”** and the **“+ESWT”** group (*p* < 0.001, each). Both the **“+ESWT”** and the **“–ESWT”** group showed statistically significantly higher values compared to the **“Untreated”** cells (*p* = 0.030 and *p* = 0.023, respectively). However, no significant differences could be observed between the cells treated and not treated with ESWT (*p* = 0.880).

### 3.7 Expression of CD163

**Fig. 6:**
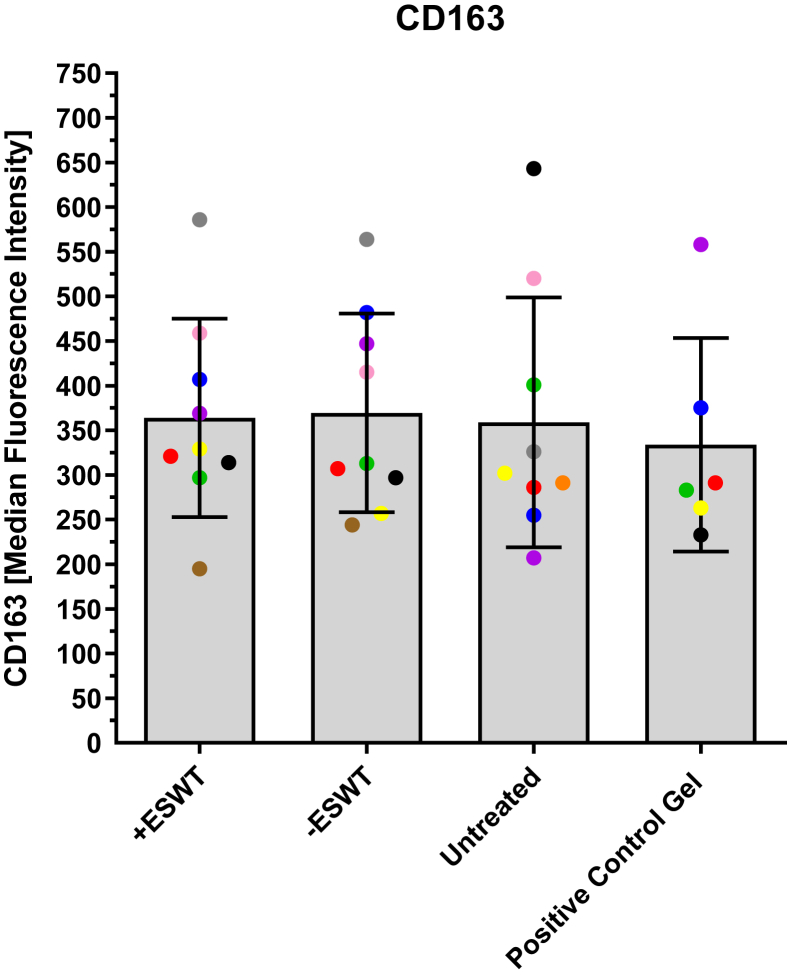
Overview of MFI values of CD163 expression measured by flow cytometry. The MFI values of the CD163 antibody in flow cytometry are presented as a boxplot (median and interquartile range) and divided into four subgroups based on the type of treatment: **“+ESWT”** (*n* = 10), **“–ESWT “**(*n* = 10), **“Untreated”** (*n* = 9), and **“Positive Control Gel”** (*n* = 6). Individual data points are represented as dots in the bar chart. Macrophages from the same patient are consistently color-coded across all images. No statistically significant differences could be observed between the groups (**“+ESWT”** vs. **“–ESWT”**: *p* = 0.919; **“+ESWT”** vs. **“Untreated”**: *p* = 0.933; **“+ESWT”** vs. **“Positive Control Gel”**: *p* = 0.624; **“–ESWT”** vs. **“Untreated”**: *p* = 0.862; **“–ESWT”** vs. **“Positive Control Gel”**: *p* = 0.565; **“Untreated”** vs. **“Positive Control Gel”**: *p* = 0.724).

Fig. 6 and Table 5 show the MFI values of CD163 expression measured by flow cytometry. The data of the individual groups differed only slightly from each other. The **“–ESWT”** group showed the highest values (369.56 ± 111.43), followed by the **“+ESWT”** group (364.11 ± 111.13) and the **“Untreated”** group (359.00 ± 139.89). The **“Positive Control Gel”** showed the lowest values (333.83 ± 119.62). There were no statistically significant differences between the groups (*p* > 0.05).

## 4 Discussion

Despite extensive research, the mechanisms behind ESWT’s anti-inflammatory effects remain unclear. Macrophages play a central role in wound healing, transitioning from pro-inflammatory M1 to anti-inflammatory M2 phenotypes, with M2 macrophages peaking around day seven post-injury. Chronic wounds disrupt this balance, with prolonged M1 activity driving inflammation and excessive M2 polarization leading to fibrosis. Targeted approaches like ESWT may enhance macrophage-driven tissue regeneration. Since the cellular mechanism behind the clinically observable anti-inflammatory has not been elucidated yet, this study aimed to investigate the impact of ESWT on macrophage polarization in a three-dimensional collagen matrix model.

However, no statistically significant differences between shockwave-treated and untreated cells for any of the investigated surface markers (CD209, CD86, CD200R, CD163) could be shown in this study. Nevertheless, the potential for polarization of M1 macrophages into M2 macrophages within the collagen gel was demonstrated through stimulation with IL-4 and M-CSF, which is well-known to induce macrophage polarization into the M2a subtype [[38], [39], [40]].

These findings are in contrast to the previous study conducted by Sukubo et al., who demonstrated ESWT to dampen the induction of the pro-inflammatory profile characterizing M1 macrophages (CD80, COX2, CCL5) and to promote the acquisition of an anti-inflammatory profile (ALOX15, MRC1, CCL18) [35]. The differing outcomes may be attributed to the model used in this study. Sukubo et al. applied shockwaves to a liquid solution within a culture flask. As mentioned earlier, it may be anticipated that a significant portion of the energy is absorbed after passing the interface. Furthermore, since evidence suggests that the cellular response to ESWT is largely determined by the complex interactions within a cellular network, involving impedance between different cell layers, intercellular connections, fluid dynamics, and chemotaxis, which are difficult to mimic *in vitro*, a key drawback of this *in vitro* model is the fluid character of cell suspension.

Furthermore, human skin exhibits anisotropic behavior during deformation, responding differently to applied forces depending on the direction due to its complex biomechanical composition [41]. One of the key physical properties frequently studied in this context is stiffness [42], which ranges widely from 360 Pa (Pa) to 160 MPa [41,43]. Therefore, our study aimed to overcome the drawback of previous *in vitro* studies by assessing macrophage polarization in a 3D collagen matrix. Here, a bovine type I collagen gel with a concentration of 2.23 mg/ml was used, corresponding to an estimated stiffness of approximately 220 Pa, near the lower limit of the reported human skin stiffness values.

To evaluate the potential polarization shift of M1 macrophages induced by ESWT, specific surface markers characteristic of M1 and M2 macrophages were analyzed. CD86, primarily expressed by pro-inflammatory M1 macrophages, served as an indicator of their proportion within the total cell population [44]. In contrast, CD209, CD200R, and CD163 were used to identify M2 macrophages. Among these, CD209 and CD163 are predominantly associated with the M2c subtype, while M2a macrophages mainly express CD200R [[45], [46], [47]]. Sukubo et al. used real-time quantitative polymerase chain reaction (qPCR) to analyze a polarization shift in their study, which might also contribute to the differing outcomes observed.

Nevertheless, the absence of phenotypic changes in macrophages following ESWT suggests that not only the stiffness of the environment but also potential influences of cell-cell interactions, which are absent in our simplified model, may play a significant role in mechanotransduction, even in this 3D collagen matrix model. Macrophages are highly dynamic cells that perform various functions depending on their phenotype, often mediated through interactions with other cells. While the role of macrophage cell-cell interactions in wound healing remains poorly studied, the results of this study suggest that communication between macrophages and other wound-healing cells may contribute to the anti-inflammatory effects of ESWT. Macrophages are particularly closely associated with fibroblasts, a relationship that significantly influences the tissue microenvironment. During an inflammatory response, fibroblasts produce M-CSF, promoting macrophage recruitment to the lesion, while activated macrophages, in turn, stimulate M-CSF production in fibroblasts [48,49]. Duffield et al. further demonstrated that the macrophage-fibroblast interaction is crucial for processes such as fibrosis, underscoring the importance of these cellular relationships in inflammation and wound healing [50]. Therefore, it remains uncertain whether the addition of other cell types alone would restore the *in-vivo*-like responsiveness to ESWT. Moreover, even with co-cultures, the mechanical stimulation profile *in vitro* may still differ significantly from the *in vivo* situation, where tissue heterogeneity, vascularization, and systemic factors play additional roles.

Thus, the lack of cell-cell interactions and the limitations of the mechanical environment in our model may both have contributed to the absence of detectable effects of ESWT on macrophage polarization and should be addressed in future research using more complex models. However, several previous studies indicated an anti-inflammatory and wound-healing effect of ESWT. This appeared to rely on mechanotransduction, where mechanical energy is converted into biological signals via cell-matrix interactions (focal adhesions) and cell-cell junctions (adherents junctions) [51,52]. These processes activate signaling cascades that influence cellular metabolism and the cell cycle, although the exact mechanisms remain incompletely understood.

Studies have demonstrated that ESWT promotes wound healing by improving blood flow through increased eNOS, VEGF, and PCNA expression [51,53,54], and modulating key signaling pathways such as ERK, FAK, Wnt/β-Catenin, and ATP/P2X7, which regulate cell differentiation and proliferation [33,51,55]. Specific anti-inflammatory effects of ESWT, including reduced levels of TNF-α, IL-1β, IL-6, COX-2, and oxidative stress markers (ROS, NADPH, and glutathione), have been linked to pathways like PI3K/AKT/FOXO1 and NF-κB/MAPK inhibition [33,[56], [57], [58], [59]]. The reduced inflammatory cytokines in treated tissue, mediated by inhibiting NF-κB and MAPK signaling pathways, suggest a potential polarization shift from M1 to M2 macrophages. However, there is currently no direct evidence to support this hypothesis.

The diverse signaling pathways identified in the literature highlight the complexity of physiological processes influenced by ESWT. Rather than a single cell type acting as the primary mediator, the effects of ESWT are likely the result of interactions among key wound-healing cells, including neutrophils, macrophages, endothelial cells, and fibroblasts. These assumptions are supported by the findings of Davis et al., who induced burn wounds in mice that were subsequently treated with ESWT 1 h later (rESWT, 200 impulses, 0.1 mJ/mm^2^, 5 Hz). While no statistically significant difference was observed in wound size, the number of infiltrating leukocytes was significantly reduced. Moreover, ESWT treatment led to reduced infiltration of neutrophils and macrophages, accompanied by a significant downregulation of neutrophil and macrophage chemoattractants, acute-phase cytokines, and key metalloproteinases involved in basement membrane degradation and remodeling [60]. Similar findings have been reported by Holsapple et al. in humans with chronic venous ulcers. The authors examined the effect of 500 impulses of ESWT at 4 Hz (0.11 mJ/mm^2^). Tissue biopsies were compared pre- and post-treatment using immunohistology. The study observed a significant reduction in wound area and macrophage counts. Interestingly, despite the overall reduction in macrophage numbers, macrophage-related markers increased, regardless of M1-or M2-like phenotypes, suggesting a complex modulation of macrophage activity by ESWT [61]. As a result, the authors conducted an *in-vitro* study to explore the effect of ESWT in a macrophage mono-culture. They observed an increase in phagocytosis, as well as in the expression of TNF, IL-1, and TGF-β. These findings are consistent with previous studies, further indicating that ESWT may enhance macrophage activity, particularly to inflammatory cytokine production [61]. Considering the proven anti-inflammatory and wound-healing effect of ESWT accompanied by the clinically observable reduction in macrophage numbers and a modulation of their activity in both, animal models and human studies, future studies employing co-culture models of these cell types in 3D matrices are needed to provide deeper insights into the cellular and immunological mechanisms underlying the effect of ESWT.

To compare the polarization potential of macrophages cultured on UpCell™ 6-well plates with those cultured in collagen gel, the “Positive Control Cell Culture Plate” group was included to rule out any influence of the collagen gel on macrophage polarization to the M2 subtype. The UpCell™ surface, made from temperature-sensitive polymers, allows cell detachment without enzymatic solutions such as Accutase® or cell scrapers [62]. At 37 °C, the surface is hydrophobic, enabling cell adhesion, while below 32 °C, it becomes hydrophilic, allowing gentle cell detachment by reducing the temperature [62]. The aim was to eliminate the potential effects of enzymatic dissociation on macrophage surface markers.

IL-4 stimulation, known to enhance CD200R expression on macrophages, resulted in a significant increase in CD200R MFI in cells cultured within collagen gel compared to those on UpCell™ plates. This suggests a greater polarization potential for macrophages in the collagen gel. Conversely, CD86, primarily expressed by pro-inflammatory M1 macrophages, was significantly higher in the “Positive Control Cell Culture Plate” group than in the gel group. However, no significant differences were observed for other markers between the two groups. Interestingly, within the “Positive Control Cell Culture Plate” group, a relatively high standard deviation in the expression of CD209 and CD86 was observed. This variability, despite identical treatment protocols, is likely attributable to inter-donor differences, as confirmed by re-analysis of the raw MFI data. Particularly, one donor’s macrophages exhibited elevated expression levels of these markers, highlighting the biological heterogeneity of primary human immune cells. For the design of future experiments, these findings indicate that the culture conditions of macrophages should be pre-evaluated for their suitability in detecting shifts in macrophage polarization. Furthermore, the results highlight the necessity of including appropriate control groups in each experiment to account for inter-patient variability. To ensure robust conclusions regarding this variability, control groups must also comprise a sufficiently large sample size, which was not the case in this study for the control cell culture plate. Overall, macrophage polarization appeared influenced by the culture environment, with collagen gel supporting higher polarization to M2 and the cell culture plate to M1 macrophages. Further studies are needed to validate these findings.

This study presents some limitations that should be acknowledged. First, in this investigation, a moderate EFD of 0.12 mJ/mm^2^ was employed. Although numerous studies have utilized this setting in cellular experiments, higher EFDs or repeated shockwave applications may produce a more pronounced effect on macrophage polarization. Additionally, reliance on a single time point (24 h post-treatment) for FACS analysis may not fully reflect the dynamic nature of macrophage polarization. Future investigations should therefore consider whether increased or repeated shockwave exposure, as well as additional or varied observation times, might yield different outcomes regarding macrophage phenotype shifts. Second, only marker expressions were measured. A functional validation of M2 macrophages, e. g. through cytokine analyses or phagocytosis assays, would offer additional information on the phenotypic shift. Third, inter-donor biological variability represents an inherent limitation of studies using primary human monocytes or macrophages. Upon re-evaluation of the raw MFI data, we identified notable deviations in marker expression in individual donors across all experimental groups, despite strict adherence to standardized protocols and the absence of technical anomalies. These findings suggest that the observed variability is likely due to donor-specific immune phenotypes. Rather than excluding such values as outliers, we chose to include all data as obtained, as they reflect the biological heterogeneity encountered in clinical settings and thus preserve the translational value of the study. Fourth, the gel itself, particularly its stiffness, may have influenced the polarization toward the M2 phenotype, although the other M2 markers were not elevated. This suggests that an effect of ESWT would have been detectable within the present study design. An important consideration, not directly assessed in this study, is whether shockwave exposure alters the structural properties of the collagen matrix itself. Such structural changes could indirectly affect macrophage polarization or activity. Variations in gel composition could affect polarization, while it is anticipated that the greatest impact would be achieved by incorporating additional cell types. Therefore, while the 3D collagen matrix model offers a more physiological environment with similar stiffness to human skin than traditional 2D cultures, it still lacks the complex interactions *in vivo*. Specifically, the absence of other key cell types that interact with macrophages in human tissues, such as fibroblasts and endothelial cells, may have limited the ability to capture the full range of macrophage responses to ESWT. Future studies should focus on developing more advanced co-culture systems incorporating multiple cell types to better simulate the *in vivo* environment. Fifth, an additional limitation concerns the propagation of shockwaves in the experimental setup. It is well established that radial extracorporeal shock waves exhibit a depth-dependent attenuation of EFD. Sawicki et al. [63] demonstrated that EFD decreases with increasing application frequency, due to incomplete projectile return and the resulting vibratory motion, with a notable decline observed at frequencies above 6 Hz. However, at the frequency employed in the present study (5 Hz), EFD remains relatively stable between 80 % and 90 % [63]. Moreover, Liu et al. [64] showed that EFD substantially diminishes when traversing biological tissues, reporting a reduction of approximately 42.5 % after passing the skin layer (ca. 0.4 mm thickness) and only 17.8 % transmission through 19.5 mm of adipose tissue. Considering the considerably thinner dimensions of the collagen gels utilized in this study (ca. 2 mm) compared to *in vivo* tissues, a lower degree of EFD attenuation is assumed. Nevertheless, spatial heterogeneity of mechanical stimulation within the gel cannot be entirely excluded and may have influenced the cellular response. Future studies should systematically assess shockwave propagation characteristics in 3D culture models with varying thicknesses to better quantify the mechanical environment experienced by cells.

## 5 Conclusions

This study aimed to investigate the effects of ESWT on macrophage polarization within a 3D collagen matrix model. Although no significant changes in macrophage polarization markers were observed following ESWT, these findings may be influenced by the simplified nature of the *in vitro* model, particularly the absence of direct cell-cell interactions with other cell types. Further research incorporating more complex co-culture systems or *in vivo* studies is required to fully elucidate the mechanisms behind ESWT’s anti-inflammatory effects and its potential for promoting tissue regeneration.

## Informed consent statement

Informed consent was obtained from all subjects involved in the study.

## Author contributions

Conceptualization, DH, NW-I, MG-L, PWK; methodology, NW-I, MG-L, KP, WB, JB; validation, DH, NW-I, MG-L, NE, JB, PWK; formal analysis, DH, MG-L; investigation, NW-I, MG-L, KP; resources, DH, WB, JB, PWK; data curation, NW-I, MG-L, KP; writing—original draft preparation, DH, NW-I, MG-L; writing—review and editing, DH, NW-I, MG-L, NE, KP, WB, JB, PWK; visualization, DH, MG-L; supervision, DH, NW-I, NE, WB, JB, PWK; project administration, DH, NW-I, WB, JB, PWK; funding acquisition, DH. All authors have read and agreed to the published version of the manuscript.

## Institutional review board statement

The study was conducted in accordance with the Declaration of Helsinki and approved by the Ethics Committee of Rhineland-Palatinate (No. 2021–15794_1 and No. 2021_16270).

## Data availability statement

Data is available at the request of the corresponding author.

## Funding

This research was funded by BiomaTiCS - Biomaterials, Tissues and Cells in Science, Mainz (internal research funding) and partly financially supported by the German Research Foundation (DFG) via CRC 1270 ELAINE.

## Declaration of competing interest

All authors were funded by the supporting societies.
